# Parkinson's disease and multiple system atrophy have distinct α-synuclein seed characteristics

**DOI:** 10.1074/jbc.RA118.004471

**Published:** 2018-11-26

**Authors:** Tritia R. Yamasaki, Brandon B. Holmes, Jennifer L. Furman, Dhruva D. Dhavale, Bryant W. Su, Eun-Suk Song, Nigel J. Cairns, Paul T. Kotzbauer, Marc I. Diamond

**Affiliations:** From the ‡ Department of Neurology, University of Kentucky, Lexington, Kentucky 40536,; the Departments of § Neurology and; ¶ Pathology and Immunology, Washington University in St. Louis, St. Louis, Missouri 63110, and; the ‖ Center for Alzheimer's and Neurodegenerative Diseases, Peter O'Donnell Jr. Brain Institute, University of Texas Southwestern Medical Center, Dallas, Texas 75390

**Keywords:** alpha-synuclein (α-synuclein), Parkinson disease, protein aggregation, biosensor, fluorescence resonance energy transfer (FRET), neurodegeneration, fibril, multiple system atrophy, prion strain

## Abstract

Parkinson's disease (PD) and multiple system atrophy (MSA) are distinct clinical syndromes characterized by the pathological accumulation of α-synuclein (α-syn) protein fibrils in neurons and glial cells. These disorders and other neurodegenerative diseases may progress via prion-like mechanisms. The prion model of propagation predicts the existence of “strains” that link pathological aggregate structure and neuropathology. Prion strains are aggregated conformers that stably propagate *in vivo* and cause disease with defined incubation times and patterns of neuropathology. Indeed, tau prions have been well defined, and research suggests that both α-syn and β-amyloid may also form strains. However, there is a lack of studies characterizing PD- *versus* MSA-derived α-syn strains or demonstrating stable propagation of these unique conformers between cells or animals. To fill this gap, we used an assay based on FRET that exploits a HEK293T “biosensor” cell line stably expressing α-syn (A53T)-CFP/YFP fusion proteins to detect α-syn seeds in brain extracts from PD and MSA patients. Both soluble and insoluble fractions of MSA extracts had robust seeding activity, whereas only the insoluble fractions of PD extracts displayed seeding activity. The morphology of MSA-seeded inclusions differed from PD-seeded inclusions. These differences persisted upon propagation of aggregation to second-generation biosensor cells. We conclude that PD and MSA feature α-syn conformers with very distinct biochemical properties that can be transmitted to α-syn monomers in a cell system. These findings are consistent with the idea that distinct α-syn strains underlie PD and MSA and offer possible directions for synucleinopathy diagnosis.

## Introduction

Fibrillar α-synuclein (α-syn)^4^ inclusion bodies define two major classes of neurodegenerative disease: Lewy body diseases (Parkinson disease and dementia with Lewy bodies) and those characterized by Papp-Lantos bodies (multiple system atrophy). These are collectively termed synucleinopathies (1–3). Synucleinopathies may progress via transcellular propagation of a unique α-syn aggregate conformer, or “seed.” This is analogous to propagation of prion protein (PrP) prions (4–6). In this model, the seed escapes one cell and acts as a template in a secondary cell to trigger further intracellular aggregation (7–10). In cell culture models, assemblies of α-syn trigger their own uptake and intracellular seeding via endocytosis mediated by heparan sulfate proteoglycans (11), and may also involve a cell surface receptor, LAG3 (12). Both *in vitro* and *in vivo* evidence supports the concept that α-syn is mobile, and that, like a prion, it can trigger development of pathology upon entry into second-order cells (13–26).

PrP prion strains are protein assemblies that consist of a defined structure, replicate faithfully *in vivo*, and produce clinically and neuropathologically heterogeneous phenotypes (27–34). Although distinct α-syn conformers have not yet been fully characterized or propagated through living systems as *bona fide* strains, multiple studies suggest they exist (35–39). For a review, see Refs. 40 and 41. This concept applies to synucleinopathies and other neurodegenerative diseases. We have found distinct conformations of tau will propagate indefinitely and produce predictable and transmissible pathology upon inoculation (34, 42, 43). Other groups have found evidence of conformation-dependent patterns of neuropathology in various β-amyloidoses (44–47).

Although Parkinson's disease (PD) and multiple system atrophy (MSA) are clinically and neuropathologically diverse (48), it is nonetheless unclear whether patients harbor α-syn strains as characterized by distinct, self-templating conformations that exhibit unique biochemical characteristics and patterns of cellular pathology. Several groups have used different methods to generate distinct populations of recombinant α-syn fibrils *in vitro* (35, 36, 49). However, it is unclear whether these assemblies consist of defined structures that replicate indefinitely upon transmission between individuals in living systems. Furthermore, the initial assemblies were created from recombinant α-syn, and it is unknown whether these conformers exist in human synucleinopathies. Watts *et al.* (26) address part of this question, reporting that homogenates from MSA brain induce seeding and transmissible pathology in α-syn transgenic mice. Other studies also reported no seeding activity in PD brain lysate, either in a biosensor cell line or in inoculated animals (37, 38). A recent study by Peng *et al.* (39) describes biochemical differences in α-syn isolated from PDD (Parkinson disease dementia) and MSA brain, however, evidence of maintenance of strain-specific characteristics through passage is still lacking. To compare and contrast PD and MSA α-syn seeds, we have used an established cell-based assay to test for self-propagating structures that exhibit unique biochemical characteristics.

## Results

### α-Syn biosensor cells detect seeding activity

**Figure 1.**
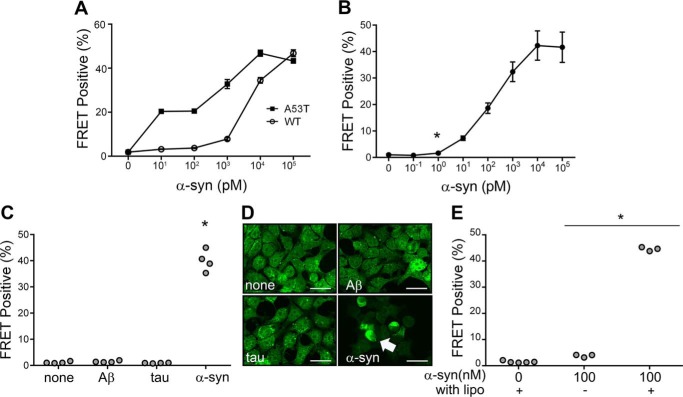
**FRET-based assay to detect α-syn seeding activity.** *A,* dose-response curves generated for seeding activity as measured by the FRET assay with α-syn (A53T)-CFP/YFP and (WT)-CFP/YFP biosensors in response to seeding with recombinant WT synuclein fibrils in transiently transfected cells. *B,* dose-response for recombinant α-syn fibrils. Stable cells expressing the α-syn (A53T)-CFP/YFP biosensor were exposed to increasing amounts of α-syn fibrils in the presence of Lipofectamine, and induced aggregation was quantified using FRET flow cytometry. Detectable conversion occurred at 1 pm fibrils (monomer equivalent); *, *p* < 0.007. *C,* α-syn-CFP/YFP biosensor cells are specific for α-syn. 10 nm α-syn fibrils produce robust seeding (**, *p* < 0.0001), but not 100 nm Aβ(1–42) or 100 nm tau fibrils. *D,* fluorescence images of induced α-syn-CFP/YFP inclusions. Images are at ×40 magnification with scale bars of 25 μm. *Arrowhead* indicates inclusion within biosensor cell. *E,* Lipofectamine enhances α-syn seed detection in the FRET biosensor assay as measured at 100 nm concentration of recombinant α-syn fibrils (*, *p* < 0.001, *t* test compared with vehicle-treated condition).

To detect α-syn seeding activity, we have previously created biosensor cell lines based on α-syn fusion to cyan and yellow fluorescent proteins (α-syn-CFP/YFP) (50, 51). We determined empirically that A53T creates the most effective biosensor for recombinant α-syn fibril detection by comparing it to WT α-syn (Fig. 1*A*). Hereafter, we refer to the α-syn (A53T) protein in the biosensor line simply as α-syn-CFP/YFP. α-Syn-CFP/YFP does not spontaneously aggregate within cells. But these cells will take up exogenous recombinant α-syn seeds that will subsequently trigger intracellular aggregation of the α-syn-CFP/YFP biosensor proteins. This can be directly visualized by epifluorescence microscopy, or quantified via fluorescence resonance energy transfer (FRET) (50, 51). In the FRET flow cytometry assay, we determined a lower limit for detection of 1 pm (monomer equivalent) recombinant α-syn fibrils (Fig. 1*B*). Exposure of cells to fibrils prepared from synthetic Aβ(1–42) and recombinant tau did not generate any signal by FRET flow cytometry, or visible inclusions via fluorescence microscopy, even at 10,000-fold over the detection limit for α-syn fibrils (Fig. 1, *C* and *D*). Although application of fibrils alone triggered seeding in the biosensor cells, addition of Lipofectamine strongly increased seeding (Fig. 1*E*). Consequently, we used this agent in our subsequent experiments.

### PD and MSA brain extracts contain α-syn seeds

**Figure 2.**
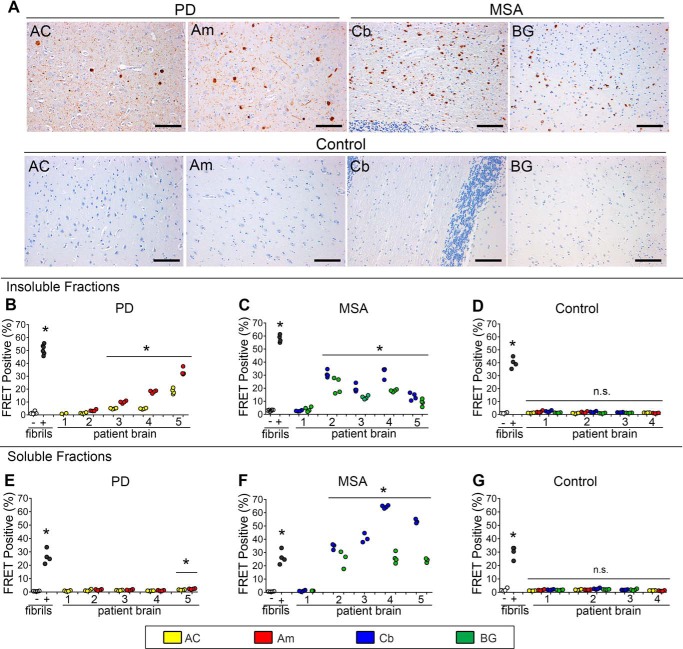
**α-Syn seeding from insoluble and soluble fractions of PD and MSA brain.** *A,* microscopy of PD, MSA, and normal control brains used in the seeding experiments. *Scale bar* = 100 μm. Patient samples are displayed as follows: PD#4 AC, PD#4 Am, MSA#4 Cb, MSA#4 BG, Control#2 AC, Am, Cb, and BG. *B,* seeding detected from insoluble fractions of PD anterior cingulate and amygdala. Controls are 10 nm recombinant fibril (+) and buffer only (−) samples. *C,* seeding detected from insoluble MSA cerebellum and basal ganglia fractions. *D,* control insoluble brain samples from matched regions exhibit no appreciable seeding. Note: no amygdala sample was available for PD1 from the autopsy bank. *, *p* < 0.004, *t* test compared with vehicle-treated condition. *E,* PD soluble fractions did not demonstrate significant seeding activity other than PD5, which is significant, *p* = 0.0003 on *t* test compared with vehicle-treated conditions (post-hoc Bonferroni correction is 0.0028 for *n* = 19 comparisons). *F,* MSA-soluble fractions contain significant seeding activity. *, *p* < 0.0006, *t* test compared with vehicle-treated conditions. *G,* soluble fractions from region-matched control samples do not demonstrate any significant seeding.

Prior reports have indicated that brain extract from MSA, but not PD, contains α-syn seeding activity (26, 37, 38). We used the α-syn-CFP/YFP biosensor cell line to test for seeding activity in postmortem brain tissue from both PD (*n* = 5) and MSA (*n* = 5) cases. We evaluated regions previously determined to have abundant α-syn pathology based on histopathological analyses by a neuropathologist (N. J. C.). In PD, the anterior cingulate gyrus (AC) and amygdala (Am) contain high levels of neuronal α-syn pathology, including Lewy bodies and Lewy neurites (Fig. 2*A*). In MSA, the cerebellum (Cb) and basal ganglia (BG) exhibit abundant inclusions (Papp-Lantos bodies) in oligodendrocytes and in white matter tracts (Fig. 2*A*). Neuropathological characteristics of all PD, MSA, and control patients are detailed in Table 1. Prior to the analysis of seeding activity using the FRET biosensor system, we sequentially extracted tissue samples according to previously described methods (52). We began with homogenization in high salt buffer without detergent, followed by ultracentrifugation at 100,000 × *g* to generate the “buffer-soluble” fraction. We then homogenized the pellet in buffers containing 1% Triton X-100, with a wash step, followed by ultracentrifugation at 100,000 × *g* to generate the “detergent-insoluble” fraction (see “Experimental procedures” for details). The detergent-insoluble fractions of both PD and MSA contained α-syn seeding activity detected by the FRET biosensor assay, with higher levels in MSA brain tissue samples (Fig. 2, *B* and *C*). Seeding activity correlated between regions for each of the patients examined, *i.e.* brains with high seeding in amygdala also had high seeding in the anterior cingulate, and vice versa. We obtained control brain samples from identical regions of patients with similar degrees of Alzheimer disease neuropathology (53) (Table 1). None of these control brain samples contained appreciable levels of α-syn seeding activity (Fig. 2*D*).

**Table 1:**
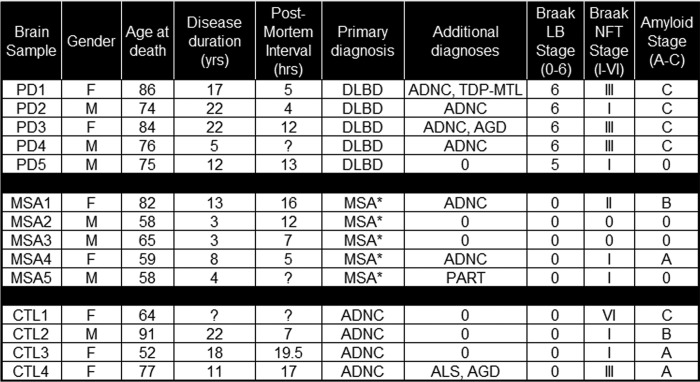
**Clinical and neuropathological characteristics of brain samples for synucleinopathy and non-synucleinopathy controls** Control samples were selected to match MSA and PD samples for a range of tau and amyloid pathology patterns. DLBD, diffuse lewy body disease; *, MSA-nigrostriatal type; ADNC, Alzheimer disease neuropathologic change; AGD, argyrophilic grain disease; TDP-MTL, TDP-43 proteinopathy in medial temporal lobe; ALS, amyotrophic lateral sclerosis; PART, primary age-related tauopathy.

Given the sensitivity of the biosensor assay, we tested for differences in the level of soluble α-syn seeding activity. We detected robust seeding activity from the soluble fractions of almost all MSA brain samples (Fig. 2*F*), whereas we detected almost none in all PD brain samples (Fig. 2*E*). Soluble fractions from controls had no appreciable seeding activity (Fig. 2*G*). Taken together, we conclude that seeding activity in PD consists predominantly of detergent-insoluble species, whereas in MSA it consists of both detergent-soluble and -insoluble forms.

**Figure 3.**
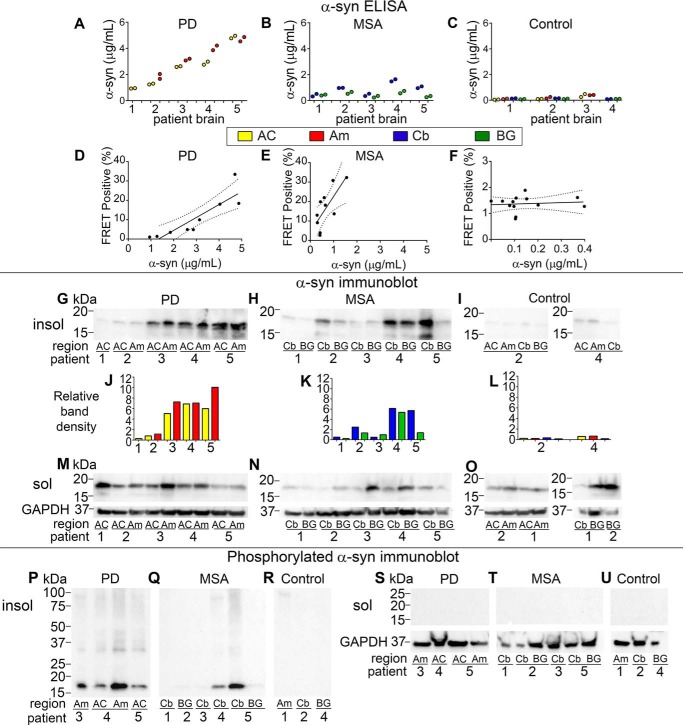
**PD and MSA total and phosphorylated α-syn levels.** *A–C*, α-syn concentration as measured by ELISA in PD-, MSA-, and Control-insoluble extracts (μg/ml). *D–F,* scatterplot of α-syn seeding activity detected by FRET assay compared with total concentration of α-syn measured by ELISA. Correlation coefficients as calculated by Spearman nonparametric two-tailed analysis: (PD, *p* = 0.0017, *r* = 0.8802; MSA, *p* = 0.0202, *r* = 0.7333; Control, *p* = 0.7343, *r* = 0.1044). *G–I,* immunoblot for total α-syn from brains of PD, MSA, and Control patient-insoluble extracts. *J–L,* relative band density as determined by comparison to equivalent concentrations of α-syn WT fibrils loaded on same blots. *M–O,* immunoblot for total α-syn from soluble extracts. *P–R,* Western immunoblot detects phosphorylated α-syn in insoluble extracts of PD and MSA but not in control insoluble extracts. *S–U,* no phosphorylated α-syn is detected in soluble extracts.

Given the observed differences in seeding activity of PD and MSA, we measured α-syn levels in the insoluble fractions by enzyme-linked immunosorbent assay (ELISA) (52). For capture, we used Syn211 antibody (Santa Cruz), which binds near the C terminus of α-syn (amino acids 121–125). For detection, we used a polyclonal antibody FL-140 (Santa Cruz). We detected higher α-syn levels in synucleinopathy cases *versus* controls. We detected higher levels of total α-syn in PD-insoluble samples than MSA-insoluble samples (Fig. 3, *A versus B*). We found the lowest level of α-syn in control groups (Fig. 3*C*).

α-Syn concentration in insoluble samples and seeding activity strongly correlated within each disease state. That is, insoluble fractions of PD with higher concentrations of total α-syn by ELISA demonstrated more seeding activity (Fig. 3*D*). There was a similar observation for insoluble fractions of MSA (Fig. 3*E*). One sample in each set of PD and MSA brains had no detectable seeding activity (Fig. 2, *B* and *C*). This correlated with lower levels of α-syn as measured in insoluble fractions by ELISA (Fig. 3, *A* and *B*) and immunoblot (Fig. 3, *G* and *H*) for both regions surveyed.

Comparing seeding activity relative to α-syn concentration between disease states, we observed higher seeding activity per ng of insoluble α-syn in MSA *versus* PD brain (*p* = 0.0202, *r* = 0.7333 for MSA, and *p* = 0.0017, *r* = 0.8802 for PD by Spearman analysis) (Fig. 3, *D* and *E*). Control samples did not show a similar correlation (*p* = 0.7343, *r* = 0.1044) (Fig. 3*F*). We verified ELISA results by immunoblot for total α-syn (Clone 42, BD Bioscience), which confirmed lower levels of total α-syn in MSA- *versus* PD-insoluble fractions, as well as minimal α-syn in control insoluble fractions (Fig. 3, *G–I*) as determined by band density relative to recombinant monomer (Fig. 3, *J–L*).

Immunoblots for α-syn in soluble fractions (utilizing Clone42 antibody) revealed relatively less total α-syn in MSA than PD (Fig. 3, *M* and *N*) and the presence of α-syn in soluble fractions from control samples, as expected (Fig. 3*O*). Given the lack of robust seeding activity seen in PD soluble samples (Fig. 2*E*), soluble seeding activity in PD did not depend on soluble α-syn concentration as measured by immunoblot (Fig. 3*M*). The one PD brain sample with detectable soluble seeding activity did not show significantly higher levels of soluble α-syn (Fig. 3*M*).

Phosphorylated forms of α-syn have been associated with pathogenicity in synucleinopathies (43). To determine whether phosphorylated α-syn was present in our fractions, and whether it correlated with seeding activity, we performed immunoblots for pSer-129 (EP1536Y, Abcam) in both insoluble and soluble fractions from MSA, PD, and control brain samples. Insoluble fractions from both PD and MSA demonstrated phosphorylation of α-syn (although PD was more consistent in this regard) (Fig. 3, *P* and *Q*), whereas insoluble control extracts and all soluble fractions, even soluble MSA extracts, did not contain detectable levels of phosphorylated α-syn (Fig. 3, *R–U*). Thus, seeding activity of α-syn derived from brain tissue did not relate to the pSer-129 phosphorylation state.

### MSA and PD seeds produce distinct inclusion morphologies

Prionopathies produce unique clinical and neuropathological syndromes depending upon the conformation, or strain, of the PrP prion (27, 29). In tauopathies, distinct tau strains correlate with specific neurodegenerative diseases, and exhibit clear differences in inclusion morphology within cultured cells that correlate with (but do not morphologically match) unique biochemical profiles and induction of different types of tauopathy *in vivo* (42). In synucleinopathies, the classic neuropathological hallmark of PD, the intraneuronal Lewy body, differs morphologically from the Papp-Lantos body seen in oligodendroglia in MSA. Given the differences we observed in the solubility of seeding activity derived from PD *versus* MSA brains, and prior reports about the failure of PD brain extracts to seed *in vitro* and *in vivo* (26, 37, 38), we hypothesized that α-syn might exhibit properties of classical prion strains. We thus tested whether seeding from PD *versus* MSA brains would produce distinct inclusion patterns in the biosensor cells.

**Figure 4.**
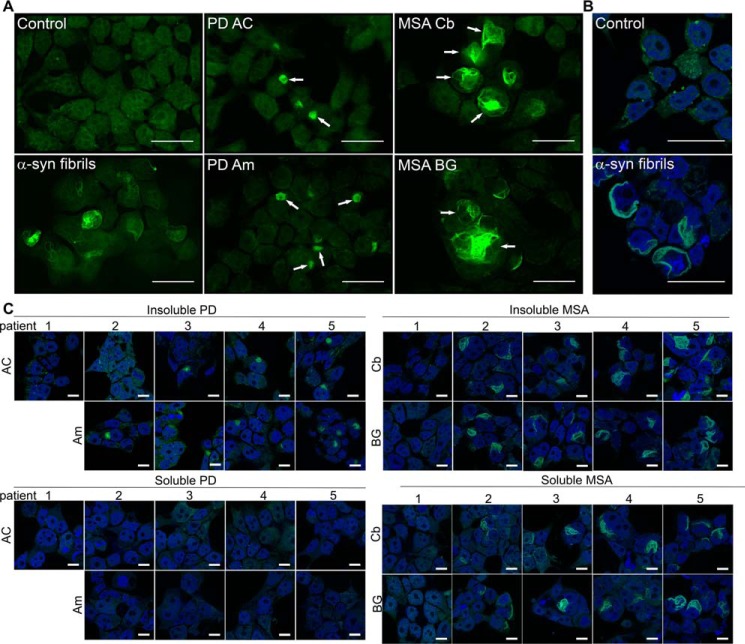
**MSA and PD induce inclusions of distinct morphology.** *A,* biosensor cells seeded with buffer (Opti-MEM) and Lipofectamine reagent only, 10 nm recombinant α-syn fibrils, or insoluble extracts from PD (patient #5) anterior cingulate, PD#4 amygdala, MSA#4 cerebellum, and MSA#5 basal ganglia. *Arrows* indicate typical aggregates within cells; circumscribed morphology from PD brain tissue and wispy morphology seen in MSA brain tissue. *Scale bars* are 25 μm. *B,* confocal pictures from cells seeded with buffer (Control) and 10 nm recombinant α-syn fibrils and counterstained with DAPI. *Scale bars* on all confocal pictures are 25 μm. *C,* confocal pictures from each brain sample demonstrate consistent morphological differences between aggregates seeded from PD-insoluble extracts and MSA-soluble and -insoluble extracts. Soluble PD fractions did not produce aggregates as expected. *Scale bars* are 10 μm.

We exposed biosensor cells to recombinant α-syn fibrils, or fractions from PD and MSA brains, and examined inclusions by fluorescence microscopy (Fig. 4*A*). We observed no visible inclusions in Lipofectamine-treated controls, whereas recombinant α-syn fibrils produced distinct inclusions. We did not observe inclusions induced by soluble PD brain fractions, as reported above. However, detergent-insoluble PD brain extract produced uniformly smaller and more circumscribed inclusions. MSA-insoluble and soluble-brain extract produced filamentous, wispy inclusions that filled the cytoplasm. The induced inclusion morphologies correlated with disease, not brain region, and soluble and insoluble material from MSA brains produced indistinguishable patterns. Inclusions were cytoplasmic, as demonstrated by confocal microscopy with a nuclear stain (DAPI) (Fig. 4, *B* and *C*). Distinct morphological characteristics were consistent across all brain samples that produced inclusion formation (Fig. 4*C*).

**Figure 5.**
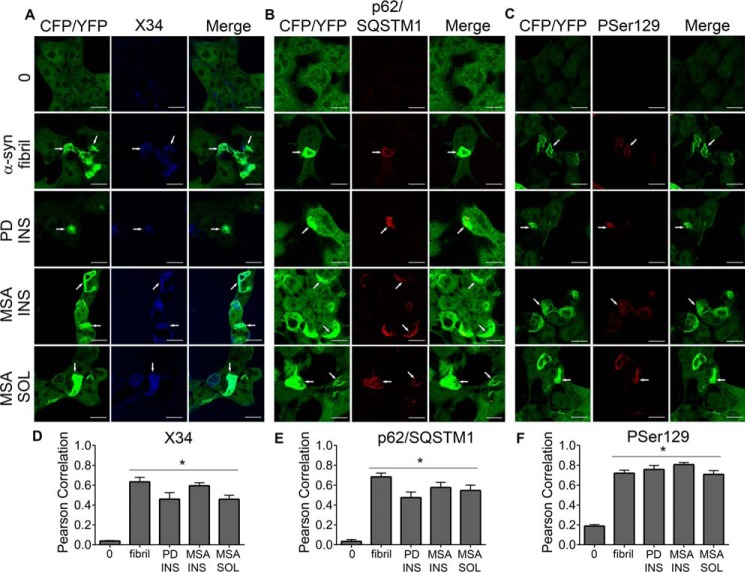
**Intracellular inclusions demonstrate colocalization with markers for aggregation.** *A,* biosensor cells seeded with PD, MSA, recombinant fibrils all demonstrated inclusions which colocalize with X34 staining. *0* is buffer-seeded, *fibril* is 10 nm recombinant α-syn fibril, *PD INS* is PD patient #4 anterior cingulate-insoluble fraction, *MSA INS* is MSA patient #5 basal ganglia-insoluble fraction, *MSA SOL* is patient #5 basal ganglia soluble fraction. *B,* inclusions were similarly positive for p62/SQSTM1 (PD INS (#4 amygdala), MSA INS (#4 basal ganglia), and MSA SOL (#4 basal ganglia)). *C,* pSer-129 staining was similarly positive (PD INS (#4 amygdala), MSA INS (#4 basal ganglia), and MSA SOL (#5 basal ganglia)). All *scale bars* are 10 μm. *Arrowheads* demonstrate colocalization of inclusions with respective markers. Pearson correlation for CFP/YFP and (*D*) X34, (*E*) p62/SQSTM1, and (*F*) pSer-129, *, *p* < 0.0001 via one-way ANOVA with Dunnett's multiple comparison to 0 (control).

To further characterize inclusions and determine whether there is similarity to pathologic α-syn found within patient brains, we performed immunocytochemistry on seeded biosensor cells. Inclusions exhibited amyloid structure based on co-localization with amyloid dye X34 (Fig. 5*A*). Inclusions were colocalized with p62/SQSTM1 staining (ab56416, Abcam), a marker for polyubiquitinated protein aggregates (Fig. 5*B*) and pSer-129 antibody P-syn/81A (Biolegend) (Fig. 5*C*). Pearson correlation coefficients indicated significant co-localization (*p* < 0.0001 one-way ANOVA with Dunnett multiple comparison test to control condition) of the X34, p62/SQSTM1, and pSer-129 stain with PD-insoluble and MSA-insoluble and -soluble aggregates (Fig. 5, *D–F*). Pearson correlation values may underestimate co-localization because brain-derived α-syn added to cells may also bind these antibodies but would not necessarily co-localize with the GFP signal.

### Induction of inclusion morphology in second-generation cells

**Figure 6.**
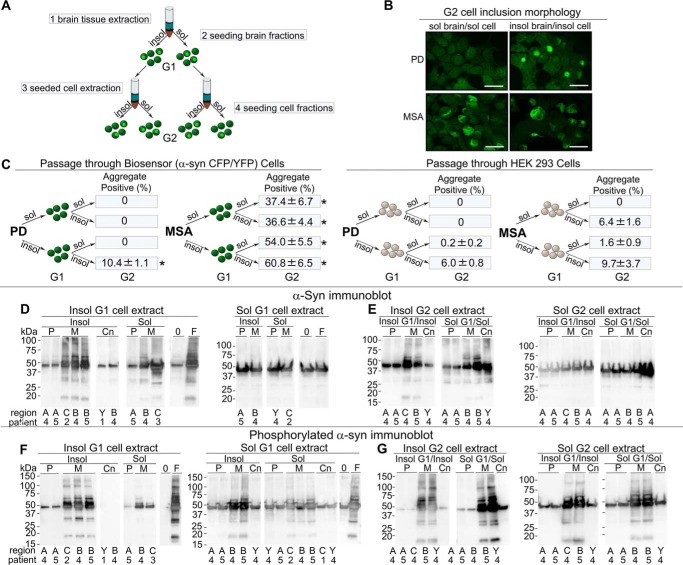
**Preservation of inclusion morphology upon conversion of second-generation cells.** *A,* experimental paradigm. Biosensor cells were seeded with insoluble or soluble extracts from MSA or PD brain. After 3 days, seeded cells were extracted and soluble and insoluble fractions obtained. These cell extracts were used to seed naive biosensor cells, and after an additional 3-day incubation, cells were assessed by fluorescence microscopy for inclusions. *B,* biosensor cells seeded with insoluble or soluble cell extract from a population of cells initially seeded with insoluble or soluble extract from PD or MSA brain. Note the preservation of inclusion morphology, and pattern of seeding that correlates with that seen from brain tissue-seeded cells. Image magnification was ×40, *scale bar* 25 μm. Images include PD sol/sol, patient #4 amygdala; PD insol/insol, #5 amygdala; MSA sol/sol, #5 cerebellum; MSA insol/insol = #4 basal ganglia. *C,* control HEK293T cells that lack α-syn overexpression or biosensor cells overexpressing α-syn (A53T)-CFP/YFP (G1), were seeded with brain extract (MSA or PD) and serially extracted after a 3-day incubation period. Soluble and insoluble cell extracts were used to seed biosensor cells (G2). After addition of exogenous seeds, biosensor cells expressing α-syn efficiently propagate seeding activity and inclusion morphology to second-generation biosensor cells (G2). HEK293T cells that lack α-syn overexpression propagate pathology inefficiently due to residual seeds carried over through them. Seeding activity in all fractions passaged more efficiently through biosensor cells than through HEK293 cells. *, *p* = 0.0005 *t* test. Results were replicated by two different methods (flow cytometry and histological cell counts) for two regions from two different PD patient brains (#4 and #5 anterior cingulate and amygdala) and two regions from two different MSA patient brains (#2 and #3, cerebellum and basal ganglia). *D* and *E,* G1- and G2-seeded cells were serially extracted and fractions run on immunoblot showing total α-syn. There are aggregated forms of α-syn in MSA-insoluble fractions, but not in PD or control samples. *P* = PD, *M* = MSA, *Cn* = Control tissue, *A* = anterior cingulate, *C* = cerebellum, *B* = basal ganglia, Y = amygdala, 0 = buffer seeded negative control, and F = recombinant fibril seeded control (10 nm). *F* and *G,* G1- and G2-seeded cells were serially extracted and α-syn assessed by Western blotting for phosphorylated forms of α-syn. Phosphorylated α-syn levels and also aggregated forms of phosphorylated α-syn were higher in MSA than in PD in both soluble and insoluble forms.

PrP prion strains always produce the same patterns of cellular pathology and disease incubation times *in vivo* (29, 31), and tau strains have similar properties (42). We thus tested whether α-syn aggregates induced in the biosensor cells by PD or MSA brain extracts would faithfully pass the same inclusion morphologies to second-generation cells. We first exposed biosensor cells to MSA or PD brain extracts. We incubated the cells (G_1_ population), for 72 h, and serially extracted the α-syn-CFP/YFP as described above in our studies of brain tissue samples. We then used these fractions to seed a naive population of biosensor cells (G2 population) using visual inspection for inclusion formation (Fig. 6*A*). Insoluble fractions of PD- and MSA-seeded biosensor cells induced inclusions with the same inclusion morphologies seen in the original seeded cells (Fig. 6*B*). Furthermore, consistent with prior seeding experiments (Fig. 2), MSA-seeded cells developed inclusions from the soluble and insoluble cell lysate fractions, whereas PD-seeded cells only developed inclusions from the insoluble material (Fig. 6*B*). To test the degree to which inclusion formation derived from induction of α-syn aggregation within biosensor cells *versus* persistence of brain-derived α-syn, we seeded HEK293T cells that lack α-syn overexpression with brain tissue in parallel with biosensor cells, prior to identical extraction. For insoluble PD material, this induced seeding at about 60% of that from lysates passaged through biosensor cells, and 17% for insoluble MSA material. Soluble MSA material passaged through control cells had 0% background (Fig. 6*C*).

Passage of proteopathic α-syn through an *in vivo* system could potentially amplify these pathogenic forms. To determine whether α-syn concentrations or phosphorylated α-syn levels changed with passage through the biosensor cells, we performed immunoblots on G1 and G2 cell extracts (Fig. 6, *D–G*). All immunoblots demonstrated a species at ∼42 kDa corresponding to the α-syn-CFP/YFP fusion proteins constitutively expressed within the biosensor cells. MSA-derived samples and to a lesser extent, PD-derived fractions, demonstrated oligomeric forms in insoluble fractions, whereas control fractions and buffer-seeded cells did not show oligomeric banding patterns (Fig. 6, *D* and *E*). Phosphorylated forms were also more prevalent in the G1 and G2 MSA-insoluble fractions. We observed an increase in phosphorylated forms of α-syn in the MSA samples over sequential passage, especially in the soluble fractions (Fig. 3, *M* and *N, versus* Fig. 6, *E* and *G*). Taken together we conclude that α-syn seeds derived from PD or MSA brain induce α-syn-CFP/YFP aggregation transmissible in biosensor cells that parallels the solubility and morphological patterns observed within human brain samples.

## Discussion

The origins of distinct α-syn-related diseases such as PD and MSA are unknown. One possibility is that distinct conformations, or “strains” of α-syn cause different disorders. Prior work has not isolated seeding activity from PD brains, however, and thus it has not been possible to directly compare PD-derived seeds to those of MSA. In this study we used a sensitive and specific FRET biosensor assay based on expression of α-syn-CFP/YFP containing the *SNCA* A53T mutation to detect α-syn seeding activity. We quantified the activity present in soluble- and detergent-insoluble brain fractions. Although both PD and MSA brains contained insoluble seeding activity, only MSA brain contained soluble activity. We further observed that PD and MSA α-syn seeds induced distinct inclusion morphologies and detergent solubility of aggregates in biosensor cells. Finally, upon transduction of brain lysates into biosensor cells, the induced aggregates exhibited detergent solubilities similar to those of α-syn seeds present in PD and MSA brains and created identical inclusion morphologies in a second generation of cells. Within the limitations of our studies, isolation and characterization of two forms of α-syn that are linked directly to PD and MSA is consistent with the existence of distinct α-syn strains.

### α-Syn biosensor assay

The biosensor assay described here used full-length α-syn (A53T) fused to CFP or YFP (α-syn-CFP/YFP), and was originally reported in a prior publication (50). We easily observed seeding of the α-syn-CFP/YFP reporter by recombinant wildtype (WT) α-syn fibrils, which has been reported *in vitro* (54). This cell-based assay allows quantification of aggregation in a population of cells by flow cytometry utilizing a distinct FRET signal, whereas single-fluorophore quantification of inclusions requires direct microscopy counts (38). Prior work has indicated that tau and α-syn biosensors are highly specific to the origin of seeds (42, 50), and in this study we also tested control samples from advanced AD cases (Braak Stage VI) that contained high levels of tau and Aβ pathology. We found no evidence of “cross-seeding” of α-syn by tau or Aβ. Although a prior report suggested that exposure of neurons in culture to α-syn fibrils “cross-seeds” tau (35), we have not observed any evidence that α-syn or tau seeds heterologous monomer in cells (42, 50).

In our cell-based assay, cationic lipid reagent (Lipofectamine) enhanced signal, presumably by increasing the efficiency of seed delivery. *In vivo* studies have noted uptake and spreading of injected forms of α-syn within the brain without additional reagents, but in cultured non-neural cells we have consistently observed a potentiating effect of Lipofectamine in seed delivery (50). This may reflect differences in cell surface glycoproteins. Nonetheless, as all samples were treated identically, we feel confident attributing our findings to differences in seeding activity in PD *versus* MSA samples.

### α-Syn seeding activity detected in both PD and MSA brains

Prior reports have indicated failure to detect α-syn seeding activity in PD brain samples (26, 37, 38). The FRET-based biosensor used here easily detected seeding activity and inclusions of distinct morphology from both PD and MSA brains. Several factors may explain these differences. First, we evaluated material from different patients and different brain regions. We examined the amygdala and anterior cingulate gyrus, regions noted to have high levels of phosphorylated α-syn, whereas prior work evaluated substantia nigra and surrounding midbrain, which typically have severe neuronal loss and low levels of synucleinopathy, at least in advanced cases. The prior studies also used phosphotungstate anion to precipitate α-syn, according to a prior published method (55), whereas we used detergent fractionation.

Immunoblots for phosphorylated α-syn from brain tissue-soluble fractions did not show detectable levels in either PD or MSA, although these same MSA samples were able to seed aggregation within the α-syn-CFP/YFP cells. This suggests that phosphorylated α-syn, whereas a reliable marker for pathology, does not necessarily correlate with seeding ability. We did see higher levels of phosphorylated α-syn after propagation in our cell system and isolation, which was especially prominent in MSA samples. PD and MSA, as well as recombinant synthetic α-syn fibril, induced aggregate formation within biosensor cells that colocalized with pSer-129 staining.

### α-Syn strains in PD and MSA

Evidence that α-syn strains account for different synucleinopathies is still incomplete. Recombinant assemblies created *in vitro* by serial seeding reactions show distinct toxicities, induction of pathology, and biochemical differences after passage *in vivo* (35, 36). However, as it is very difficult to amplify a single fibrillar conformation *in vitro*, these α-syn assemblies might have been of heterogeneous composition, and do not necessarily reflect those produced during the life of the patient. Prior work has described α-syn seeding activity of MSA brain extracts, but left in question whether PD extract actually contains any seeding activity (26, 37, 38). Our work is consistent with a recent study that has found biochemical-based differences between Sarkosyl-extracted α-syn from PDD and MSA brain (39). However, there is still a lack of convincing evidence that strain-specific properties are maintained after passage through *in vitro* or *in vivo* systems. In this study we used a biosensor cell line to determine that brain extracts from both PD and MSA contain α-syn seeding activity. Both diseases had seeding activity in detergent-insoluble brain fractions, but MSA also had such activity in the detergent-soluble fraction. This suggests a possible explanation for the failure of several prior studies to detect seeding activity in PD brain. Although we detected lower levels of total α-syn in MSA *versus* PD by two different biochemical methods, we observed much more robust seeding activity in MSA. This was most evident in soluble fractions. ELISA and immunoblot data both indicate that total levels of α-syn do not necessarily predict seeding ability of samples, especially in MSA-soluble samples. Seeding ability of α-syn derived from brain also does not seem dependent on pSer-129 α-syn, suggesting that while this post-translational modification is an important pathological marker, it may not be necessary for seeding ability of α-syn.

We propose that the seeding activity in MSA may be inherently distinct from PD. This is consistent with other studies that have only found seeding activity in MSA (26, 37, 38). PD and MSA brain extracts also produced different inclusion morphologies in biosensor cells. Distinct inclusion morphologies imply, but do not prove, that underlying fibril structure differs. However, inclusion morphology across brain regions and patient samples for PD *versus* MSA was strikingly consistent. These different morphologies were associated with the same detergent solubility profiles observed in human brain. We also observed faithful preservation of inclusion morphologies upon inoculation of a new population of cells.

Immunoblots of α-syn demonstrate insoluble α-syn-CFP/YFP in fractions from cells seeded with soluble PD brain extract and soluble control brain extract, although corresponding inclusions are not detected in these fractions by flow cytometry FRET signal. One possibility is that a low level of aggregation occurs in unseeded α-syn-CFP/YFP cells that is present in the insoluble fraction but is below the level of detection on the FRET assay. A second explanation would be that a portion of insoluble α-syn does not fluoresce in this system and is not detected on FRET flow cytometry. In this last case, FRET may underestimate the amount of aggregation that occurs from α-syn seeding. A third possibility would be carryover of monomeric forms of α-syn in the insoluble fraction, in which case the insoluble fraction would not solely contain aggregated forms of α-syn. This is partially supported by low levels of insoluble α-syn seen in immunoblots from control brain tissue, which would not be expected to have aggregated forms of α-syn and does not have FRET activity. It should also be noted that the presence of insoluble α-syn does not necessarily equate with seeding activity, and the seed-competent component may only comprise a small fraction of the total insoluble α-syn pool. Further experiments will be necessary to define the species that induce α-syn aggregation.

Although it seems likely that α-syn strains exist, no study, including this one, has indefinitely propagated patient-derived seeds from cell-to-cell, or mouse-to-mouse, or fully characterized α-syn strains from MSA *versus* PD. Indeed, we have failed to propagate distinct α-syn conformers in clonal lines as we have done for tau (42, 43) because aggregate-containing clones lose the aggregated state over time. To confirm the existence of *bona fide* α-syn prion strains, it will be necessary to test whether distinct structures stably propagate through living systems and produce consistent pathology, as do tau and PrP. Taken together with prior studies by others, however, our findings are very consistent with the existence of different α-syn strains in PD and MSA.

## Experimental procedures

### Recombinant α-syn purification and fibrillization

Recombinant α-syn monomer was produced as previously described (52). Purified recombinant α-syn fibrils were generated from incubation of monomer (2 mg/ml) for 72 h at 37 °C with shaking (1000 rpm) in buffer (20 mm Tris-HCl, pH 8, and 100 mm NaCl). After centrifugation at 15,000 × *g* for 15 min, the concentration of monomer in the supernatant was determined by bicinchoninic acid (BCA) assay (Micro BCA Protein Assay Kit, ThermoScientific) and the measured decrease in monomer was used to determine the concentration of fibrils in the pellet.

### Preparation of Aβ(1–42) and tau fibrils

Synthetic Aβ(1–42) and tau fibrils were produced according to published methods (52, 56). 1 mg of synthetic Aβ(1–42) was dissolved in 50 μl of DMSO. Then, 925 μl of distilled deionized H_2_O with 25 μl of 1 m Tris-HCl, pH 7.6, was added and the reaction mixture was incubated for 30 h at 37 °C with shaking at 1,000 rpm in an Eppendorf Thermomixer. To determine the concentration of fibrils, the fibril reaction mixture was centrifuged at 15,000 × *g* for 15 min to separate fibrils from monomer. The concentration of Aβ(1–42) monomer in the supernatant was determined in a BCA protein assay along with a BSA standard curve. The measured decrease in monomer concentration was used to determine the concentration of Aββ(1–42) fibrils in the 30-h fibril reaction mixture.

Purified recombinant tau monomer (300 μg/ml) in 20 mm Tris-HCl, pH 8.0, 100 mm NaCl, 25 μm low molecular weight heparin, 0.5 mm DTT was incubated for 48 h at 37 °C with shaking at 1,000 rpm in an Eppendorf Thermomixer. To determine the concentration of fibrils, the fibril reaction mixer was centrifuged at 15,000 × *g* for 15 min to separate fibrils from monomer. The concentration of tau monomer in the supernatant was determined in a BCA protein assay along with a BSA standard curve. The measured decrease in monomer concentration was used to determine the concentration of tau fibrils in the 48-h fibril reaction mixture.

### Generation of stable cell lines

Stable cell lines were generated as previously described (50). HEK293T cells were plated at 150,000 cells/well and subsequently transduced with α-syn (A53T)-CFP and α-syn (A53T)-YFP lentiviral constructs. After 48–72 h cells were resuspended in flow buffer and sorted by FACS (Sony Biotechnology) to select a polyclonal population of dual α-syn-CFP/YFP–expressing cells with a donor:acceptor molar ratio of 1:1. Median fluorescence intensity ratio of CFP:YFP was (1:2.5) in this CFP/YFP population. Populations of cells expressing either α-syn (A53T)-CFP or α-syn (A53T)-YFP were also isolated and selected as single positive controls. Dual positive cells were expanded, and subsequently diluted onto 10-cm^2^ dishes. Single colonies were selected with cloning cylinders (Bel-Art Products) and expanded. All FRET and fluorescence microscopy experiments in this paper utilized a monoclonal cell line as the α-syn-CFP/YFP biosensor.

### Preparation of soluble and insoluble fractions from postmortem brain tissue

The Movement Disorders Center Neuropathology Core, Washington University, St. Louis, MO, provided clinically and neuropathologically well-characterized postmortem frozen brain tissue (57). Routinely, microscopy was performed on the left hemibrain and biochemistry was performed using the right hemibrain. α-Syn-immunoreactive inclusion bodies were observed only in PD and MSA utilizing phosphor-dependent anti-α-syn immunohistochemistry (Cell Applications, San Diego, CA) and #64 (Wako, Osaka, Japan, respectively). Frozen tissue was prepared as previously described (52, 58). Tissue was first dissected into small pieces (∼1 mm square) using a scalpel and a cutting board cooled with solid CO_2_. Tissue was then weighed and serially extracted with a series of buffers, using a Dounce homogenizer (Kontes) for homogenization. A ratio of 3 ml of buffer to 1 g of tissue was maintained throughout the extraction process. Initial homogenization was performed in high salt buffer (50 mm Tris-HCl, pH 7.4, 750 mm NaCl, 5 mm EDTA) with protease inhibitors (Sigma), followed by centrifugation at 100,000 × *g* for 20 min at 4 °C. Supernatant was removed and used as the buffer “soluble” fraction for further experiments. The pellet was homogenized in 1% Triton X-100 in high salt buffer with protease inhibitors, followed by centrifugation at 100,000 × *g* for 20 min at 4 °C. The pellet was extracted with 1 m sucrose and 1% Triton X-100 in high salt buffer. The myelin component and supernatant were removed, and the pellet was then washed twice with Tris-buffered saline (TBS), resuspended in TBS, with protease inhibitor (Sigma), aliquoted, and frozen at −80 °C and used as the detergent “insoluble” fraction.

### FRET seeding assay

Monoclonal biosensor cells were plated in 96-well plates at 35,000 per well and grown overnight. The following day, recombinant fibrils or brain lysate fractions were sonicated for 3 min at 65A (Qsonica 700) at 4 °C. Samples were prepared in Opti-MEM (Gibco) with 1 μl of Lipofectamine 2000 (Invitrogen) to a total volume of 20 μl/well and incubated at room temperature for 30 min. Samples were then added dropwise to wells. Technical quadruplicates were performed for each sample. For samples testing brain lysate, 5 μl of soluble or insoluble material was added per well. Unless otherwise specified, incubation was for 72 h. Cells were then trypsinized and transferred to 96-well round-bottom plates, fixed in 4% paraformaldehyde (Electron Microscopy Services) in PBS for 10 min, then centrifuged and resuspended in flow buffer (1 mm EDTA and 1% FBS in Hank's balanced salt solution). Finally, they were assessed for aggregate load using a MACSQuant VYB (Miltenyi) as previously described (50). Analysis was performed using FlowJo v10 software (TreeStar).

Transient transfection experiments were conducted as described above with the exception of an initial plating step into 12-well plates at 285,000 cells/well followed by transfection with 4 μl of Lipofectamine per well with 250 ng/well of α-syn WT- or mutation A53T-CFP/YFP constructs in a FM5 plasmid, a modified version of the plasmid as described in Ref. 59. This was followed by replating of cells into 96-well plates and transfection of recombinant fibrils or brain lysate 1 day later.

### Cell immunofluorescence

Glass coverslips were coated with poly-d-lysine hydrobromide (Sigma) overnight at room temperature in 24-well plates, then washed 3 times with sterile water and allowed to air-dry for 2 h. Cells were plated at 50,000 cells/well. Cells were transduced the next day with 10 nm fibrils or 35 μl of insoluble or soluble brain fractions with Lipofectamine (3 μl/well). For experiments comparing seeding in HEK cells not overexpressing α-syn with biosensor cells expressing α-syn-CFP/YFP, after initial seeding as above, cells were incubated for 72 h and subsequently lysed by sonication and serially extracted through the same buffer and centrifugation steps as above. BCA assay was performed on the soluble and insoluble cell extracts and protein concentrations were calculated. Equivalent concentrations of lysate from cells not overexpressing α-syn and lysate from biosensor cells expressing α-syn-CFP/YFP, were added when seeding the naive population of biosensor cells. After 72 h, cells were fixed with 4% paraformaldehyde (EMS) for 15 min and washed with Dulbecco's PBS three times. They were then mounted on slides using Fluoromount G with DAPI (Southern Biotechnology).

For photographs, cells were imaged on a Nikon Eclipse TE2000-U fluorescence microscope using a Nikon Plan Fluor ×40/0.75 objective. Metamorph software (Molecular Devices) was used with Nearest Neighbors algorithm for deconvolution. For analyses of aggregate percentages in HEK and biosensor cells, cells were imaged on a Zeiss microscope and fields throughout each slide were selected by randomly visualizing fields with >20 DAPI+ nuclei at ×60. DAPI was used to quantify the total number of cells, and aggregates were counted under a FITC fluorescent channel (Chroma FITC filter set: excitation 480 ± 20 nm, emission 535 ± 25 nm and Chroma DAPI filter set: excitation 350 ± 25 nm, emission 460 ± 25 nm).

### Immunofluorescence and X34 staining

Cells were plated on glass coverslips precoated with poly-d-lysine, seeded with brain tissue or recombinant fibrils, and fixed with 4% paraformaldehyde as above, then rinsed 3 times for 5 min each in PBS. For immunofluorescent stains, cells were incubated in 0.1% Triton X-100 in PBS for 15–20 min at room temperature. For SQSTM1/p62 (Abcam) cells were blocked in 3% normal goat serum (Invitrogen) for 20 min and then incubated in primary antibody 1:100 with 1.5% normal goat serum overnight at 4 °C. For pSer-129 staining, cells were blocked in 3% FBS with 3% BSA for 1 h at room temperature. Primary antibody was P-syn/81A (Biolegend), 1:5000, and cells were incubated in 3% BSA overnight at 4 °C. Subsequently, they were washed 3 times for 5 min each with PBS then incubated with secondary antibody (either anti-mouse Alexa Fluor 647 or anti-rabbit Alexa Fluor 647) 1:500, Jackson ImmunoResearch) for 1 (SQSTM1/p62) or 2 h (P-syn/81A) at room temperature. After another three washes for 5 min each, the coverslips were mounted on slides with Fluoromount G (Southern Biotech). For X34 staining, cells were incubated in 0.25% Triton X-100 in PBS for 30 min at room temperature. X34 (Sigma) was dissolved in a staining buffer of 60% PBS, 38% ethanol, and 0.02 m NaOH to a stock concentration of 1 mm. This was further diluted in PBS to a 2 μm concentration and cells were stained at room temperature for 15 min. Samples were rinsed three times for 5 min each in PBS then mounted in Fluoromount (Southern Biotech). For confocal microscopy, cells were imaged on Nikon C2 Plus confocal laser system coupled to a Nikon Eclipse Ti microscope. Images were acquired utilizing a Nikon PlanApo oil immersion objective with numerical aperture of 1.45 and resolution of ×100 with virtual zoom for the images was set to 2.74. Identical intensities and settings were maintained during imaging of each series. Pearson correlation was derived by taking >5 different images per condition and defining regions as cells. In conditions with aggregates, only cells with inclusions were analyzed. Pearson values were calculated by the NIS elements colocalization function.

### ELISA

ELISA analysis of α-syn was performed as previously described with minor modifications (52). Costar 96 half-well plates were incubated with Syn211 antibody (Santa Cruz Biotechnology, Inc.) diluted 1:200 in carbonate coating buffer and incubated at 4 °C overnight. Plates were washed five times with PBS/Tween 20 (0.05%) (PBS-T) and blocked with 2% BSA/PBS for 2 h at 37 °C. After five more washes with PBS-T, samples were diluted in PBS-T (with 0.6% SDS) and protease inhibitor (Sigma), and then incubated overnight at 4 °C with rocking. Standard curve samples were prepared in equivalent concentrations of SDS from recombinant α-syn monomer. All standards and samples were run in duplicate. After 5 washes of PBS-T, polyclonal capture antibody FL-140 (Santa Cruz Biotechnology, Inc.) was diluted 1:200 in 0.05% BSA in PBS-T and added to each well. Samples were incubated for 2 h at 37 °C. After 5 washes in PBS-T, biotinylated anti-rabbit secondary horseradish peroxidase antibody (Jackson ImmunoResearch) was diluted 1:800 in 1% BSA/PBS-T and incubated in the dark for 1.5 h at room temperature. After 5 washes, the plate was developed with super slow 3,3′5,5′-tetramethylbenzidine (Sigma) and absorbance was determined at 650 nm on a Bio-Tek Synergy 2 plate reader after 45 s of shaking.

### Immunoblotting

For brain tissue assessment, samples were sonicated for 3 min at 65A (QSonica S-4000). After a pulse spin, for insoluble samples, 4× sample buffer (Bio-Rad) with β-mercaptoethanol was added to each sample (soluble samples were diluted 1:1 with sample buffer, to reduce distortion from high salt buffer). Samples were heated at 95 °C × 5 min, then quenched on ice. After a pulse spin, samples were loaded and run on SDS-PAGE using a 4–20% Tris glycine TGX gel (Bio-Rad) at 100 V × 1.5 h. After three 5-min rinses in transfer buffer, they were transferred onto nitrocellulose, 0.2 μm (Bio-Rad), at 150 mA for 3.5 h on ice. Ponceau was used to visualize transfer. Blots were washed three times for 5 min with T-TBS (TBS with 0.1% Tween 20) and blocked for 30 min with 5% milk in T-TBS. Primary antibody incubation was done overnight at 4 °C in 5% milk in T-TBS with the following (anti-synuclein Clone 42 BD Bioscience, pSer-129 EP1536Y Abcam). After three 5-min washes in T-TBS, blots were incubated in secondary anti-mouse horseradish peroxidase (Invitrogen) at 1:10,000 in 5% milk with T-TBS. After another wash as above, blots were visualized with ECL plus substrate (Pierce) and imaged on a Bio-Rad imager with quantification by Image Lab.

For serial seeding and extraction experiments (G1 and G2 cell extraction), α-syn-CFP/YFP cells were plated on 12-well plates at a density of 260,000 cells/well and seeded 18 h later as described above. Three days after seeding, cells were collected and lysed by sonication for 1 min at 65A, then serially extracted through high salt buffer, 1% Triton X-100 and a series of TBS washes interspersed with centrifugation at 100,000 × *g* as described above. Instead of Dounce homogenization, trituration was used to resuspend the pellet after each spin. BCA quantification of the soluble and insoluble cell extracts was performed. Equal amounts of extract (30 μg for insoluble fractions and 10 μg for soluble fractions) were prepared and diluted in TBS with protease inhibitor (Sigma) to equal volumes, then seeded onto G2 cells plated at equivalent density in 12-well plates. These were then similarly collected and serially extracted into soluble and insoluble fractions as above.

### Statistics

All analyses, unless otherwise noted, were performed using Prism software (GraphPad). Student's *t* tests were performed with post-hoc Bonferroni corrections for the number of comparisons per set. *p* values of <0.05 were considered to be statistically significant. Results are expressed as scatter plots. For Pearson correlation statistics, one-way ANOVA analysis was conducted with multiple comparison test to the control condition. Graphs are displayed as mean ± S.E.

## Author contributions

T. R. Y., P. T. K., and M. I. D. conceptualization; T. R. Y., B. B. H., J. L. F., D. D. D., N. J. C., P. T. K., and M. I. D. resources; T. R. Y., B. B. H., J. L. F., B. W. S., E.-S. S., and N. J. C. data curation; T. R. Y., B. B. H., J. L. F., D. D. D., B. W. S., E.-S. S., P. T. K., and M. I. D. formal analysis; T. R. Y. and M. I. D. supervision; T. R. Y., B. B. H., and N. J. C. investigation; T. R. Y. and M. I. D. writing-original draft; T. R. Y. and M. I. D. project administration; T. R. Y., B. B. H., J. L. F., D. D. D., N. J. C., P. T. K., and M. I. D. writing-review and editing; B. B. H., J. L. F., B. W. S., E.-S. S., P. T. K., and M. I. D. methodology.

## Footnotes

4 The abbreviations used are:

α-syn: α-synuclein
PD: Parkinson's disease
PDD: Parkinson's disease dementia
MSA: multiple system atrophy
PrP: prion protein
CFP: cyan fluorescent protein
Aβ: amyloid β
Cb: cerebellum
BG: basal ganglia
AC: anterior cingulate gyrus
Am: amygdala
FBS: fetal bovine serum
BCA: bicinchoninic acid
PBS-T: PBS Tween 20
DAPI: 4′,6-diamidino-2-phenylindole
BCA: bicinchoninic acid
TBS: Tris-buffered saline
ANOVA: analysis of variance.
